# Morbidity amongst South African Hajj pilgrims in 2023—a retrospective cohort study

**DOI:** 10.1038/s41598-024-62682-z

**Published:** 2024-06-01

**Authors:** Ozayr Mahomed, Mohammed Nasir Jaffer, Salim Parker

**Affiliations:** 1 Discipline of Public Health Medicine, University of KwaZulu-Natal Nelson Mandela School of Medicine, George Campbell Building, Howard College Campus, Durban, South Africa; 2 Sports Physician, 68 Florida Street Ravensmead, Cape Town, 7480 South Africa; 3 Division of Infectious Diseases and HIV Medicine, Department of Medicine, University of Cape Town, Cape Town, South Africa

**Keywords:** Public health, Medical research

## Abstract

South Africans are not accustomed to the dry arid climate and sweltering heat in Saudi Arabia. We conducted a retrospective cohort study to identify the common health conditions pre-Hajj, during the 5 days of Hajj and on return to South Africa from Hajj amongst the 2023 pilgrims. A QR code and a mobile link to a self-administered questionnaire was sent to all 3500 South African pilgrims. Five hundred and seventy-seven pilgrims returned the completed surveys. Mean age of the participants was 48 years (SD 12) with a higher female representation (3:2). Forty eight percent (279) had pre-existing chronic conditions. Forty five percent (259) reported being ill during their stay in the Kingdom, 20% (115) reported having an illness during the main 5 days, whilst 51% (293) reported having an illness within 7 days of returning to South Africa. Only six pilgrims were admitted to hospital after their return home. Respiratory tract linked symptoms were the most frequently reported (95% pre Hajj and 99% post Hajj). Participants who reported having a chronic condition (AOR 1.52 95% CI 1.09–2.11) and engaging in independent exercising prior to Hajj (AOR 1.52–1.07–2.10) were at an increased likelihood of developing an illness within 7 days of returning home. Post travel surveillance swabs to identify potential pathogens that the returning pilgrims are incubating should be explored to guide further interventions.

## Introduction

A mass gathering is a planned or spontaneous event where the number of people attending could strain the planning and response resources of the community or country hosting the event^1^. Mass gathering events include public celebrations, concerts, sporting events, religious gatherings, or political rallies. Mass gathering events are increasing in frequency and are growing in complexity^2^.

Pilgrimage or Religious mass gatherings occur repeatedly in the Christian, Muslim and Hindu faiths^3^ and usually take place in a defined location, over a limited period, observing specific rituals at a specific time-interval, using a prescribed dress code, and attendance by pilgrims from multiple countries. Overcrowding, climatic conditions, physical stress, or risks due to pre-existing medical conditions expose pilgrims to significant health risks, such as the spread of infectious diseases^4^.

The Hajj is one of the largest annual mass gatherings^5^ attracting more than two million Muslim pilgrims from all areas of the globe. The proportion of pilgrims attending from each country is decided based on the proportion of the global Muslim population living in every country^4^. Most countries, including South Africa, have a Hajj mission, authorised by the Saudi government to facilitate the distribution of the Hajj visas with each country implementing their own selection criteria.

Muslims in South Africa make up 1.6% of the total population of the country, with the highest proportion living in the Western Cape^6^. South Africa received an allocation of 2500 Hajj visas from the Saudi Arabian Government in 2023, and this was increased to 3500 about 2 months prior to the commencement of Hajj. The South African Hajj and Umrah Council (SAHUC) allocates the Hajj visas based on a scoring system using a first come, first serve basis for prospective pilgrims scoring the maximum points. Pilgrims utilise an accredited tour operator and travel to the Kingdom between 2 and 6 weeks prior to the main Hajj rituals.

Hajj 2023 occurred during the Saudi summer season with recorded temperatures during the Hajj range between 37 and 45 °C^7^. South Africans are not accustomed to this dry arid climate and sweltering heat. The age profile of South African pilgrims ranges from early adulthood, middle aged and elderly with varying degrees of pre-Haj physical conditions. The extreme heat, overcrowding and long duration of the trip increases the pilgrim’s risk of exacerbation of the current pre-existing conditions and/or developing infections and injuries.

In this study we conducted a retrospective cohort study to identify the common health conditions during the trip to Saudi Arabia, during the 5 days of Hajj and on return to South Africa amongst the 2023 Hajj pilgrims. Although a number of previous studies have reported on hajj related morbidity and mortality, the South African cohort is usually younger than most pilgrims attending from other regions of the world. In addition, a number of prospective Hajj pilgrims participate in a 12 week fitness program prior to embarking on the Hajj journey. In addition to adding to the already existing database of Hajj related illnesses, we wanted to determine if the fitness program provided any added benefit.

## Methods

### Study design and population

The study was a retrospective cohort study. The target population included all 3500 South African Hajj pilgrims that were accredited and undertook the Hajj 2023 journey. Pilgrims were distributed across all provinces in South Africa.

### Sample size

A total of 3500 South African pilgrims have been accredited. We used Epi Info 7 Stats Cal to determine the minimum sample size. We varied the expected frequency of illness to 25%, 50% and 80%. With 5% margin of error. The sample size required varied between 230 for 80% frequency, 267 for 25% frequency and 384 for 50% frequency. It was our intention to obtain close to maximum participation.

### Data sources

The investigators developed a questionnaire to collect demographic characteristics (age, gender, marital status, profession, and level of education, smoking, self-perception of fitness level, weight status, duration of Hajj journey), pretravel chronic diseases such as diabetes, hypertension, chronic kidney disease, chronic obstructive pulmonary diseases, and malignancies), participation in a fitness programme prior to Hajj, illnesses prior to the 5 day pilgrimage during the 5 day pilgrimage and within 14 days of returning home, symptoms, duration and treatment including any hospitalization.

### Data collection

During registration process all the pilgrims provided a mobile number for registration purposes. The South African Hajj and Umrah council as well as travel operators were requested to send the electronic link to all pilgrims on their respective databases. The link contained the participant information sheet and informed consent document. After agreeing to participate the participants were directed to the questionnaire. The questionnaire was self-administered and was less than 10–15 min duration.

### Ethical approval

All tenets of the Declaration of Helsinki were complied with, and the study obtained ethical clearance from the Biomedical and Research Ethics Committee of the University of KwaZulu Natal (BREC: 00005678/2023).

### Data analysis

Statistical analysis was performed using IBM Statistical Software for Data Science (STATA) version 18, (IBM Corp. Armonk, NY). Age was the only continuous variable and was computed as mean ± SD, whilst all other categorical variables were recorded as percentages. Initially a descriptive analysis was conducted. Bivariate analysis was performed to calculate unadjusted odds ratio for all sub-categories for demographic variables. All the baseline variables (age category, gender, smoking status, marital status, health status prior to Hajj, body weight, vaccination status, attendance at a fit for Hajj program, number of kilometres that the person can walk, Hajj package duration and presence or absence of a chronic condition) were included in the model to assess the association between demographic factors, attendance of an exercise program and the development of illness prior to and post Hajj using a *v*alue of 0.05.

## Results

Table 1 summarizes the demographic characteristics of the participants. Five hundred and seventy-seven pilgrims returned the completed surveys. Forty seven percent (269) of the participants purchased a 3–4-week package, whilst a further 45% (262) purchased a 5–6-week package. The mean age of the participants was 48 years (SD 12) with both males and females having similar age distribution. There was a higher female representation with a sex ratio of 3 females for every two males. Most of the pilgrims assessed their health to be excellent (121, 21%) or good (286, 50%) prior to the Hajj journey. Thirty two percent (184) were overweight and 18 (3%) were obese. Although 33% (189) attended a formal exercise program prior to Hajj, 60% (346) indicated that they exercised prior to Hajj. Thirty three percent (193) were able to walk between 5 and 10 km comfortably, whilst 162, 28%) could walk more than 10 km comfortably. Forty eight percent (279) had one or more chronic condition that they were being treated for (Table 1).

**Table 1:** Frequency distribution of the demographic profile of the participants.

Variables	Category	Number	Percentage (%)
Gender	Females	347	60
	Males	230	40
Age (category)	18–29 years	27	5
	30–39 years	140	24
	40–49 years	157	27
	50–59 years	146	25
	60–69 years	90	16
	> 70 years	17	3
Age	Mean age (SD)	48 (12)	
	Median age (IQR)	47 (38–57)	
Smoking status (current)	No	493	85
	Yes	84	15
Marital status	Single	31	5
	Married	475	82
	Divorced	40	7
	Widowed	31	5
Health status prior to Hajj	Poor	20	3
	Average	150	26
	Good	286	50
	Excellent	121	21
Weight status prior to Hajj	Underweight	6	1
	Normal	369	64
	Overweight	184	32
	Obese	18	3
Vaccination status before Hajj	No	35	6
	Yes	542	94
Attendance at a fitness program	No	388	67
	Yes	189	33
Exercise before Hajj	No	231	40
	Yes	346	60
Kilometres can walk	< 2.5 km	75	13
	2.5–5 km	147	25
	5–10 km	193	33
	> 10 km	162	28
Hajj package	0–2.5 weeks	46	8
	3–4 weeks	269	47
	5–6 weeks	262	45

**Figure 1:**
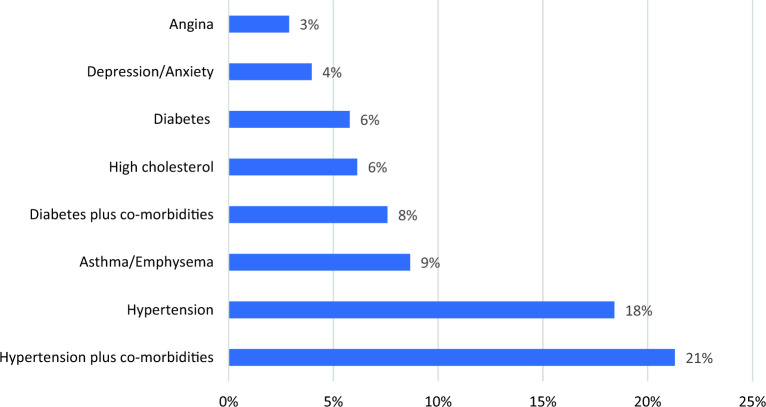
Chronic conditions reported by pilgrims-2023.

Forty eight percent (279) of the pilgrims reported having been diagnosed with a chronic disease prior to the embarking on Hajj. The eight most co0mmon chronic conditions are displayed in Fig. 1. Hypertension as a single diagnosis (18%) or hypertension with other co-morbidities (21%) were the most common chronic condition. Nine percent of the pilgrims had asthma/emphysema.

**Figure 2:**
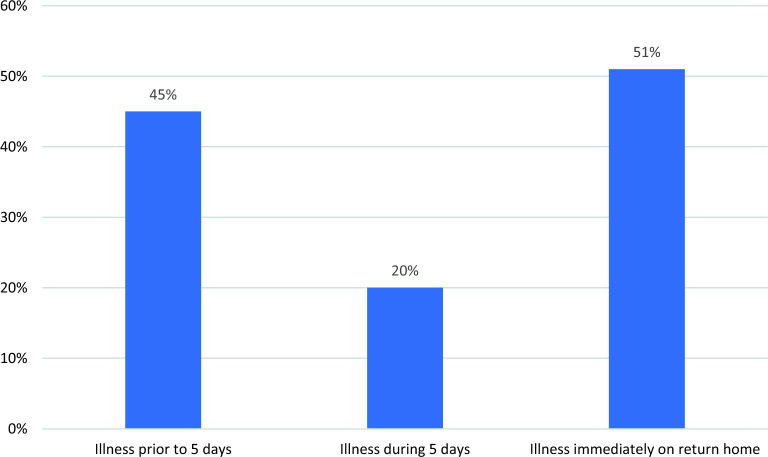
Proportion of pilgrims reporting having an illness pre-during and post Hajj-2023.

Forty five percent of pilgrims (259) reported being ill during their stay in the Kingdom. Of those, 65% (168/259) experienced a single illness during their stay, whilst 23% (60)60 (23%), 7% (18) 4% (12)12 (4%) experienced two, three and four or more episodes respectively. During the main 5 days of the pilgrimage 20% (115) reported having an illness, whilst 51% (293) reported having an illness within 7 days of returning to South Africa (Fig. 2). Only six pilgrims reported being admitted to hospital after their return home.

The most frequent symptoms and signs reported by the pilgrims that were ill pre (95%)-during (71%) and post Hajj (99%) were respiratory tract linked and included throat infections, congested sinuses, influenza, and chest infections. During the main 5 days of the pilgrimage 9% (10/115) had gastro-enteritis, whilst 12% (14/115) experienced foot problems.

Participant’s who were 60–69 years of age (UOR 2.67 95% CI 1.10–6.50); attendance of an exercise program prior to Hajj (UOR 1.54 95% CI 1.09–2.18) and participants with chronic conditions (UOR 1.55 95% CI 1.10–2.19) showed a significantly greater likelihood to report an illness after returning home Widowed participants (UOR 0.26 95% CI 0.19–0.75) and participants with excellent health status before Hajj (UOR 0.34 95% CI 0.12–0.96) were significantly less likely to report an illness after returning home (Table 2).

**Table 2:** Univariate bi-variate and multi-variate analysis of demographic profile of Hajj 2023 pilgrims and developing an illness.

Variables	Category	No illness on return home	Report illness after returning home	Unadjusted odds ratio	95% confidence interval	*p* value	Adjusted odds ratio	95% confidence interval	*p* value
Gender	Females	170 (29%)	177 (31%)	Reference			Reference		
	Males	115 (20%)	115 (20%)	0.96	0.67–1.35	0.81	0.94	0.64–1.36	0.72
Age (category)	18–29 years	17 (3%)	10 (2%)	Reference			Reference		
	30–39 years	80 (14%)	60 (10%)	1.28	0.55–2.98	0.56	1.41	0.58–3.41	0.45
	40–49 years	75 (13%)	82 (14%)	1.86	0.80–4.32	0.15	2.25	0.92–5.53	0.08
	50–59 years	67 (12%)	79 (14%)	2	0.86–4.67	0.11	2.45	0.97–6.20	0.06
	60–69 years	35 (6%)	55 (19%)	2.67	1.10–6.50	0.03	3.7	1.37–10.02	0.01
	> 70 years	11 (1%)	6 (1%)	0.93	0.26–3.28	0.91	1.37	0.34–5.47	0.66
Smoking status (current)	No	235 (41%)	258 (45%)	Reference					
	Yes	50 (9%)	34 (6%)	0.62	0.37–1.01	0.05	0.54	0.32–0.89	0.02
Marital status	Single	11 (2%)	20 (3%)	Reference					
	Married	236 (41%)	239 (41%)	0.56	0.26–1.19	0.13	0.42	0.19–0.94	0.04
	Divorced	17 (3%)	23 (4%)	0.74	0.28–1.95	0.55	0.43	0.15–1.24	0.12
	Widowed	21 (4%)	10 (2%)	0.26	0.19–0.75	0.01	0.14	0.13–0.45	0.001
Health status prior to Hajj	Poor	6 (1%)	14 (2%)	Reference					
	Average	74 (13%)	76 (13%)	0.44	0.16–1.21	0.11	0.65	0.22–0.94	0.04
	Good	138 (24%)	148 (26%)	0.46	0.17–1.23	0.12	0.62	0.15–1.24	0.12
	Excellent	67 (12%)	54 (9%)	0.34	0.12–0.96	0.04	0.49	0.43–0.45	0.001
Weight status prior to Hajj	Underweight	5 (1%)	1 (0.17%)	Reference					
	Normal	182 (32%)	187 (32%)	5.13	0.59–44.40	0.14	4.14	0.45–37.87	0.21
	Overweight	90 (16%)	94 (16%)	5.22	0.60–45.57	0.13	3.77	0.40–35.30	0.25
	Obese	8 (1%)	10 (2%)	6.25	0.60–64.86	0.13	3.64	0.32–41.92	0.3
Vaccination status before Hajj	No	22 (4%)	13 (2%)	Reference					
	Yes	263 (46%)	279 (48%)	1.8	0.84–3.96	0.1	1.57	0.74–3.29	0.24
Attendance at a fitness program	No	198 (34%)	190 (33%)	Reference					
	Yes	87 (15%)	102 (18%)	1.22	0.85–1.76	0.26	0.96	0.64–1.44	0.84
Engaiging in independent exercise before Hajj	No	129 (22%)	102 (18%)	Reference					
	Yes	156 (27%)	190 (34%)	1.54	1.09–2.18	0.01	1.52	1.07–2.10	0.02*
Kilometres can walk	< 2.5 km	31 (5%)	44 (8%)	Reference					
	2.5–5 km	81 (14%)	66 (11%)	0.57	0.33–1.01	0.05	0.61	0.34–1.12	0.11
	5–10 km	88 (15%)	77 (13%)	0.84	0.49–1.44	0.53	0.97	0.53–1.77	0.92
	> 10 km	85 (15%)	77 (13%)	**0.64**	0.37–1.11	0.11	0.81	0.43–1.54	0.52
Hajj package	0–2.5 weeks	21 (4%)	25 (4%)	Reference					
	3–4 weeks	134 (23%)	135 (23%)	0.85	0.45–1.58	0.6	0.81	0.42–1.55	0.52
	5–6 weeks	130 (23%)	132 (23%)	0.85	0.45–1.60	0.62	0.75	0.38–1.48	0.41
Chronic condition	No	163 (28%)	135 (23%)	Reference					
	Yes	122 (21%)	157 (27%)	1.55	1.10–2.19	0.008	1.52	1.09–2.11	0.01*

Significant values are in bold.

*p < 0.05.

After multi-variate analysis participants who were 60–69 years of age (AOR 3. 7 95% CI 1.37–10.02) and participants that were engaging in independent exercise prior to Hajj (AOR 1.52 95% CI 1.07–2.10) and participants with chronic conditions (AOR 1.52 95% CI 1.09–2.11) showed a significantly greater likelihood to report an illness after returning home. Widowed participants (UOR 0.14 95% CI 0.13–0.45) and participants with excellent health status before Hajj (AOR 0.34 95% CI 0.12–0.96) and participants who smoked (AOR 0.54 95% CI 0.32–0.89) were significantly less likely to report an illness after returning home.

## Discussion

Fifty one percent (293) of the current cohort of Hajj 2023 pilgrims reported acquiring an illness within 7 days of returning from pilgrimage. Although, a similar incidence (52%) was reported among Australian from Greater Sydney, New South Wales, Hajj travellers aged 18 years or older in 2015^8^, the incidence exceeds the 40% (365/915) reported amongst South African pilgrims attending Hajj in 2017^9^. In contrast to the relatively higher post Hajj illness, 45% of pilgrims (259/577) reported being ill during their stay in the Kingdom, with 30% (168/577) experiencing a single illness during their stay, which was lower than the 65.1% (596/916) reported amongst the 2017 South African Hajj cohort^9^ and the 74% amongst the 2015 Australian Hajj cohort^8^.

Globally the high frequency of respiratory infections during the Hajj and post Hajj transmission is well described^10^, and respiratory tract infections are the most frequently reported disease among South African pilgrims returning from the Hajj^11^. In our study 95% and 99% of the ill pilgrims during and post Haj reported respiratory symptoms. This is much higher than the 70% pre-Hajj and 80% post Hajj reported for the 2017 South African Hajj cohort^9^, however the pre Hajj incidence is similar to the Malaysian study that reported a 95.2% incidence of respiratory symptoms amongst pilgrims during their stay in Saudi Arabia^12^. Previous estimates indicate that 33% of Hajj pilgrims develop a respiratory infection^13^ and in our study 43% and 50% of the pilgrims reported respiratory tract symptoms prior to and post Hajj respectively.

Age, gender, marital status, baseline weight status and perception of health status, smoking vaccination status, duration of Hajj package, participation at a fitness for Hajj 12 week program prior to Hajj and distance that a person was able to walk did not show any statistical association with developing an illness on return from Hajj. Pilgrims with chronic conditions were significantly more likely to develop a post Hajj illness (AOR 1.52 95% CI 1.09–2.11). Previous studies have indicated that hypertension and diabetes are associated with increased risk of respiratory tract infections^14,15^. In the current study 47% of the pilgrims had a chronic disease with hypertension with or without co-morbidities (39%) being the most common chronic disease followed by diabetes with/without co-morbidities (14%).

Vaccination uptake was high amongst the pilgrim with 85% (490) receiving yellow fever, quadrivalent meningococcal vaccine and 44% receiving the Influenza vaccine. In addition, 9% of the pilgrims were vaccinated against pneumococcal disease). Although vaccinations may have reduced the severity of the illness, it did not confer significant protection against developing a respiratory illness post Hajj.

The high incidence of respiratory illnesses during the pilgrims stay in Saudi Arabia can be attributed to crowding, fatigue and the extreme climatic conditions that facilitate droplet and aerosol spread of particulates^16^. We postulate that international travel and seasonality have additionally contributed to the high incidence of respiratory tract infections post Hajj.

Most pilgrims are exhausted post the 5-day rituals and many return immediately home. Exhaustion compounded by the long duration of travel back to South Africa that includes a bus trip from Mecca to Jeddah, extended waiting in a crowded setting at the Hajj terminal, and exposure to re-circulated air from other pilgrims that maybe incubating infections can increased risk of transmission. Secondly, the Hajj in Saudi Arabia was during the peak summer season and on return the pilgrims were exposed to peak winter weather in South Africa and the environmental conditions may have increased the incidence of respiratory tract episodes and symptoms^17^.

Our study indicates that pilgrims who did independent exercise prior to Hajj were more likely to develop a respiratory infection. We did not assess whether the independent exercise performed prior to Hajj were moderate or strenuous. Athletes and individuals involved in heavy training programmes and/or prolonged bouts of exercise appear to have an increased risk of contracting upper respiratory tract infections^18^. Previous studies have shown that endurance athletes who experience significant stress and sleep deprivation Increase the risk of infection^19^. The external factors such as duration of travel, extensive pathogen exposure, sleep disruption, journey related stress, and change in diet and nutritional habits during the Hajj journey may have compounded the effect on the immune system and increased their risk for upper respiratory tract infection^20^.

Smoking was a surprisingly protective factor against reporting an illness immediately post Hajj. These findings maybe due to the lower prevalence of pilgrims reporting to be smokers rather than a postulated ‘smoker’s paradox^21^. Research has shown that compared with non-smokers, currently smoking was associated with higher rates of severe infectious respiratory diseases such as pneumonia, other acute lower respiratory tract infection and influenza^22^.

### Study limitations

Although, we reached the minimum sample size required, out sample represents only 17% of the total pilgrims and the findings may not be generalizable. We note that majority of the participants were from the Western Province and Gauteng which are the two Provinces that experience harsher winters and therefore inflating the incidence of respiratory tract infections. Most of the respondents were early to middle aged adults and therefore it is likely that morbidity is under-represented. Furthermore, recall bias may have affected the participants reporting of symptoms that may have occurred prior to, during and after Hajj and resulted in an under-estimate of the true prevalence of symptoms presented in this study.

## Conclusion

Majority of South African Hajj pilgrims in 2023 experienced respiratory related illness within 7 days of their return. Pilgrims with pre-existing chronic diseases of lifestyle were at an increased risk of developing an illness on return from the pilgrimage. Vaccinations against Influenza and pneumococcal disease may have decreased severity but did not decrease the incidence. It is important that the consistent observation of non-pharmaceutical interventions such as wearing of masks in crowded environments, frequent hand washing and social distancing is emphasised. In addition, it maybe necessary to conduct post travel surveillance swabs to identify potential pathogens that the returning pilgrims are incubating. These findings may direct appropriate antibiotic prescription and recommendation on future travel vaccines for Hajj.

## Data Availability

The datasets generated during and/or analysed during the current study are available from the corresponding author on reasonable request.

## References

[1] World Health Organisation (2019). Emergencies: WHO's Role in Mass Gatherings.

[2] Molloy M, Browne C, Horwell T, VanDeVelde J, Plunkett P (2019). Anatomy of a “mass” mass gathering. Prehosp. Disaster Med..

[3] Gautret P (2015). Religious mass gatherings: Connecting people and infectious agents. Clin. Microbiol. Infect..

[4] Almehmadi M, Alqahtani JS (2023). Healthcare research in mass religious gatherings and emergency management: A comprehensive narrative review. Healthcare.

[5] Fteiha B, Abul Al-Rub T, Schwartz E, Lachish T (2021). Morbidity among Arab–Israeli and Palestinian Hajj Pilgrims: A prospective study. Am. J. Trop. Med. Hyg..

[6] Statistics South Africa. *Census 2022* (Statistics South Africa, 2023).

[7] Badrek-Alamoudi AH (2023). Cellulitis in Hajj Pilgrims: Role of environmental temperature and population size of pilgrims as a contributory factor. Cureus.

[8] Alqahtani AS, Tashani M, Heywood AE, Almohammed ABS, Booy R, Wiley KE, Rashid H (2020). Tracking Australian hajj pilgrims’ health behavior before, during and after hajj, and the effective use of preventive measures in reducing hajj-related illness: A cohort study. Pharmacy.

[9] Mushi A, Yassin Y, Khan A, Alotaibi B, Parker S, Mahomed O, Yezli S (2021). A longitudinal study regarding the health profile of the 2017 South African hajj pilgrims. Int. J. Environ. Res. Public Health.

[10] Hoang VT, Dao TL, Ly TDA, Belhouchat K, Chaht KL, Gaudart J, Mrenda BM, Drali T, Yezli S, Alotaibi B (2019). The dynamics and interactions of respiratory pathogen carriage among French pilgrims during the 2018 Hajj. Emerg. Microbes Infect..

[11] Parker S, Hoosen AA, Feldman C, Gamil A, Naidoo J, Khan S (2018). Respiratory infections due to Streptococcus pneumoniae and the influenza virus in South Africans undertaking the Hajj. South. Afr. J. Infect. Dis..

[12] Deris ZZ, Hasan H, Sulaiman SA, Wahab MS, Naing NN, Othman NH (2009). Preference of treatment facilities among Malaysian Hajj pilgrims for acute respiratory symptoms. Saudi Med. J..

[13] Mansouri F, Nemat Khorasani E, Izadi M (2013). Respiratory tract infection among Hajj pilgrims. Int. J. Travel Med. Glob. Health.

[14] Zekavat SM, Honigberg M, Pirruccello JP, Kohli P, Karlson EW, Newton-Cheh C, Zhao H, Natarajan P (2021). Elevated blood pressure increases pneumonia risk: Epidemiological association and mendelian randomization in the UK Biobank. Med.

[15] Hsia CC, Raskin P (2005). The diabetic lung: Relevance of alveolar microangiopathy for the use of inhaled insulin. Am. J. Med..

[16] Alzeer AH (2009). Respiratory tract infection during Hajj. Ann. Thorac. Med..

[17] Walsh, N. P. *et al.* Position statement. Part two: Maintaining immune health. *Exerc Immunol Rev.* **17**, 64–103 (2011). 21446353

[18] Jones A, Davison G, Zoladz JA (2019). Exercise, immunity, and illness. Muscle and Exercise Physiology.

[19] Nieman DC, Wentz LM (2019). The compelling link between physical activity and the body's defense system. J. Sport Health Sci..

[20] Walsh NP, Gleeson M, Shephard RJ, Gleeson M, Woods JA, Bishop NC, Fleshner M, Green C, Pedersen BK, Hoffman-Goetz L (2011). Position statement. Part one: Immune function and exercise. Exerc. Immunol. Rev..

[21] Muhammad Shariq U, Tariq Jamal S, Muhammad Shahzeb K, Urvish KP, Izza S, Jawad A, Ankur K, Erin DM (2021). Is there a smoker’s paradox in COVID-19?. BMJ Evid. Based Med..

[22] McGeoch LJ, Ross S, Massa MS, Lewington S, Clarke R (2023). Cigarette smoking and risk of severe infectious respiratory diseases in UK adults: 12-year follow-up of UK biobank. J. Public Health.

